# Enhanced representation of spectral contrasts in the primary auditory cortex

**DOI:** 10.3389/fnsys.2013.00021

**Published:** 2013-06-19

**Authors:** Nicolas Catz, Arnaud J. Noreña

**Affiliations:** Laboratory of Adaptive and Integrative Neurobiology, Fédération de recherche 3C, UMR CNRS 7260, Université Aix-MarseilleMarseille, France

**Keywords:** tinnitus, synaptic depression, inhibition, mach bands, artificial scotoma, hearing loss

## Abstract

The role of early auditory processing may be to extract some elementary features from an acoustic mixture in order to organize the auditory scene. To accomplish this task, the central auditory system may rely on the fact that sensory objects are often composed of spectral edges, i.e., regions where the stimulus energy changes abruptly over frequency. The processing of acoustic stimuli may benefit from a mechanism enhancing the internal representation of spectral edges. While the visual system is thought to rely heavily on this mechanism (enhancing spatial edges), it is still unclear whether a related process plays a significant role in audition. We investigated the cortical representation of spectral edges, using acoustic stimuli composed of multi-tone pips whose time-averaged spectral envelope contained suppressed or enhanced regions. Importantly, the stimuli were designed such that neural responses properties could be assessed as a function of stimulus frequency during stimulus presentation. Our results suggest that the representation of acoustic spectral edges is enhanced in the auditory cortex, and that this enhancement is sensitive to the characteristics of the spectral contrast profile, such as depth, sharpness and width. Spectral edges are maximally enhanced for sharp contrast and large depth. Cortical activity was also suppressed at frequencies within the suppressed region. To note, the suppression of firing was larger at frequencies nearby the lower edge of the suppressed region than at the upper edge. Overall, the present study gives critical insights into the processing of spectral contrasts in the auditory system.

## Introduction

The main goal of the central auditory system is to organize the acoustic environment into a coherent auditory scene, namely to detect, localize, discriminate, segregate and identify the multiple sources composing a sound mixture (Bregman, 1990; Darwin, 1997; Griffiths and Warren, 2004; Shamma and Micheyl, 2010). At the initial steps of processing, the auditory system can rely on the fact that sensory objects are composed of spectral cues such as spectral edges or contours where the stimulus energy reaches a maximum or changes abruptly over frequency (Moore and Glasberg, 1983; Assmann and Summerfield, 2004; Palmer and Shamma, 2004). While spectral peaks produced by vocal tract resonances are known to play an important role for indentifying conspecific vocalizations, in speech for example (Darwin, 1984; Assmann and Nearey, 1987; Henry et al., 2005), spectral troughs or notches produced by the head-related transfer function are also recognized as being critical for localizing sound sources on the sagittal plane (Carlile et al., 2005; Grothe et al., 2010).

A critical issue in auditory neuroscience is how the central auditory system represents acoustic stimuli, in particular the frequency-specific information that is critical for organizing the auditory scene. One possibility is that the central representation mirrors that found in the peripheral cochlear nerve, whereby peaks and troughs in the spectral envelope of the acoustic stimulus could be represented by peaks and troughs in the firing rate of neurons along the tonotopic axis (Sachs and Young, 1979; Blackburn and Sachs, 1990; Silkes and Geisler, 1991; Poon and Brugge, 1993; Conley and Keilson, 1995; May et al., 1998; Recio and Rhode, 2000). However, this peripheral “rate-place” representation has significant limitations. First, the rate-place representation strongly depends on the frequency resolution of the auditory system. In particular, the spectral decomposition carried out by the cochlea tends to smooth the internal representation of the spectral envelope of complex acoustic stimuli (Moore and Glasberg, 1987; Baer et al., 1993). Second, the rate-place representation of the spectral envelope in the cochlear nerve is degraded at high levels of stimulation where the firing rate of cochlear neurons tend to saturate and/or the auditory filters broaden (Sachs and Young, 1979; Glasberg and Moore, 2000; Palmer and Shamma, 2004; Oxenham and Simonson, 2006). Finally, the peak-to-valley ratio of the rate-place representation is further decreased by the presence of background noise, which fills in the spectral valleys (Baer et al., 1993; Assmann and Summerfield, 2004).

The limitations of the cochlear nerve representation suggest that the central auditory system may have developed a strategy to overcome them, in particular to enhance the representation of spectral edges and spectral contrasts (energy ratios between adjacent peaks and valleys). The visual system, for instance, is thought to rely heavily on this mechanism. This is suggested, in particular, by the phenomenon of “Mach bands” which refers to illusory bands perceived at the spatial boundaries where the stimulus luminance changes abruptly over space (Von Bekesy, 1967, 1969a,b). While there have been some attempts to investigate this issue in audition (Von Bekesy, 1967; Carterette et al., 1969; Houtgast, 1972), it is still unclear whether a related process plays a significant role in this modality. Interestingly, however, some psychoacoustic phenomena are consistent with a mechanism enhancing spectral edges. For example, neural enhancement at spectral edges may account for the pitch induced by noise bands at their spectral edges (Small and Daniloff, 1967; Bilsen, 1977), and for the dominant role played in pitch perception by the lowest and highest partials of a harmonic complex, especially when the low-numbered (resolved) partials are removed from the complex (Dai, 2000; Moore and Gockel, 2011).

The aim of the present study is to investigate the sensory representation of the stimulus spectrum in auditory cortex, and in particular whether the representation of spectral edges is enhanced. This was accomplished by employing acoustic stimuli composed of multiple pure tones of various frequencies and presented randomly over time. These stimuli can be thought as mimicking acoustic environments with different spectral profiles when time-averaged over a few hundreds of milliseconds. Importantly, the fact that this particular stimulus was composed of a mixture of tone pips with non-synchronous onsets allowed for estimating the spectro-temporal receptive fields of cortical neurons for different time-averaged spectral envelopes (deCharms et al., 1998; Blake and Merzenich, 2002; Valentine and Eggermont, 2004; Norena et al., 2008). The present study extends earlier work where the dependence of the spectro-temporal acoustic context on cortical neurons were investigated (Gourévitch et al., 2009).

## Methods

### Animal preparation

The care and use of animals used in this study were approved by the Animal Care Committee of Bouches du Rhones, France (# A 13-504). Ten guinea pigs weighing between 300 and 800 g were used for this study. All animals were deeply anesthetized with the administration of 50 mg/kg of ketamine hydrochloride (Imalgene 1000) and 3 mg/kg of xylazine (Rompun 2%), injected intramuscularly; 0.1 ml of Atropine methyl nitrate and an analgesic (Tolfedine) were also administered. Throughout the experiment, anesthesia was maintained with half the dose of ketamine and xylazine administrated every hour. The tissue overlying the frontal lobe was opened and two screws were fixed to the top of the skull (on the antero-posterior axis) with dental cement, and used to fixate the animal's head. The tissue overlying the right or left side of the skull above the temporal lobe was removed. The skull was opened and the dura was cut back to expose the primary auditory cortex (AI). We used the location of the electrodes (Wallace et al., 2000) as well as the characteristic frequency of the neurons to ensure that the electrodes were located in AI (i.e., progression of best frequencies across electrodes). The body temperature was maintained at 37°C with a thermostatically controlled heating blanket. After the experiment, a lethal dose of sodium pentobarbital was administered.

#### Acoustic stimulation

Stimuli were generated in MATLAB and transferred to an RP2.1-based sound delivery system (Tucker Davis Technologies). Acoustic stimuli were presented in a sound booth room from a headphone (Sennheiser HD595) placed 10 cm in front of the ear contralateral to the cortex where the recordings were carried out. The amplitude of each tone pip was adjusted to the transfer function of the sound delivery system so that they were presented at the desired level in dB SPL.

**Figure 1:**
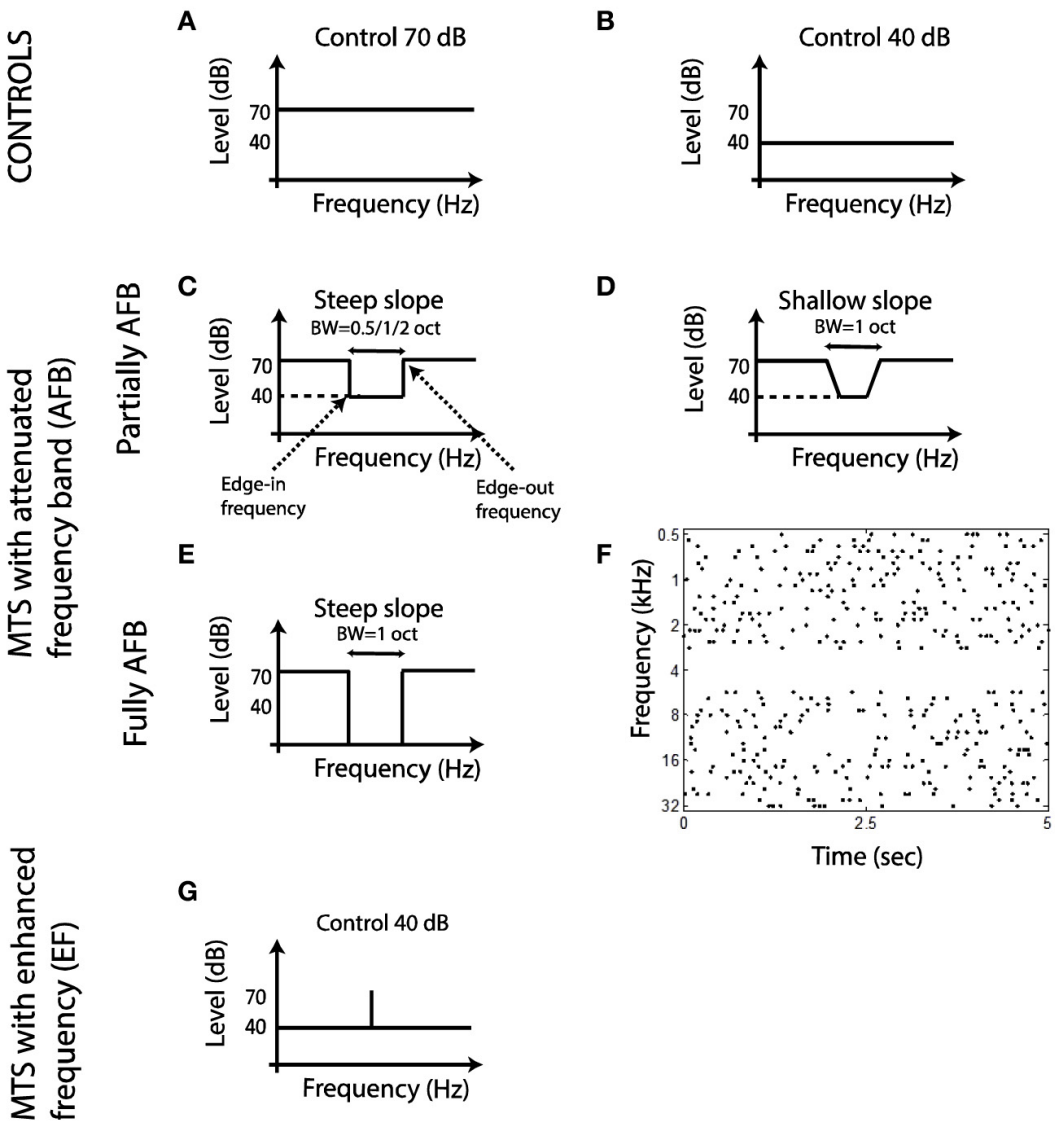
**Schematic representation of the acoustic stimuli used in the present study. (A,B)** Long-term spectrum of control stimuli. **(C–E)** Long-term spectrum of multi-tone stimuli with an AFB. **(F)** Time onsets of tone pips for a stimulus with a fully AFB (AFB centered on 4 kHz, time sample: 5 s). **(G)** Long-term spectrum of a multi-tone stimulus with an enhanced frequency.

Spectro-Temporal Receptive Fields (STRFs) were obtained from a 180-s multi-tone pip stimuli (Figure 1F) (deCharms et al., 1998; Blake and Merzenich, 2002; Valentine and Eggermont, 2004; Norena et al., 2008; Gourévitch et al., 2009). Tone pips (49 frequencies, 8 frequencies per octave covering 6 octaves) were presented randomly over time (independent Poisson process for each frequency with a rate of 2 Hz and a 50-ms dead time designed so that tones of the same frequency did not overlap in time). Tone pips of different frequencies could overlap in time. The envelope of the tone pips is given by γ(*t*) = (*t*/4)^2^ *e*^−*t*/4^ with *t* in milliseconds (stimulus duration is 50 ms, maximum amplitude is reached at 8 ms). The average rate of tone pip presentation was around 16 Hz/octave (considering the number of tone frequencies present per octave, along with the average rate of presentation of each). Control STRFs were obtained from multi-tone stimuli with tone pips presented at 70 dB SPL (ctrl-70) or 40 dB SPL (ctrl-40) (Figures 1A,B). In the attenuated frequency band (AFB) conditions, all pure tones were presented at 70 dB, except those corresponding to the frequency band of the AFB where pure tones were omitted or presented at 40 dB SPL, producing a large or moderate spectral contrast, respectively (Figures 1E and C,D). The frequencies immediately outside of the AFB were called the edge-out frequencies, while the frequencies immediately inside of the AFB were called the edge-in frequencies (Figure 1C). The bandwidth of the AFB was varied (0.5, 1, and 2 octaves). The slope of the spectral contrast (transition in dB/oct between the edge-in frequency and the edge-out frequency) was 240 dB/oct in all conditions (namely 30-dB difference between the edge-in frequency and the edge-out frequency, except in one AFB condition (with 1-oct bandwidth) where the slope was 80 dB/oct until the level of the tone pip frequencies around the center frequency of the AFB was 40 dB (Figures 1C,D). The center frequency of the AFB was set as follows. First, the BF for each cortical site was derived from the control stimulus (ctrl-70). The center frequency of the AFB was then set to the BF of a given cortical site. Cortical responses were obtained for all stimulus conditions (different widths, slopes and depths) for that specific center frequency of the AFB. Once a set of recordings was completed, another set of recordings was carried out with a different AFB stimulus (centered on the BF of another cortical site). And so on for all cortical sites with a significant STRF (see below). One notes that as we recorded from many cortical sites simultaneously, the BFs could correspond to the center frequency of the AFB, one edge frequency of the AFB, or a remote frequency from the AFB. An additional stimulus condition was investigated which consisted of multi tone pips where all pure tones were presented at 40 dB SPL, except at one frequency which was presented at 70 dB SPL (Figure 1G). Some example sound files are provided in the supplemental material.

#### MUA and LFP recording procedure

Each set of recordings was obtained with 1 array of 16 electrodes (Alpha-Omega Eng, Nazareth, Israel) arranged in an 8 by 2 pattern with 0.25 mm electrode separation within the long row and 0.5 mm separation between rows. The electrodes had impedances between 0.8–1.4 MOhm. The array was manually advanced using a Narishige microdrive into primary auditory cortex (according to the location provided by Wallace et al., 2000). The signals were then amplified 10,000 times with filter cutoff frequencies set at 2 Hz and 5 kHz. The amplified signals were processed by a TDT-System three multichannel data acquisition system. Multi-unit activity was sampled at 24,414 Hz and was extracted from the 300-Hz high-pass filtered signal. Local field potentials (LFPs) were sampled at 1061 Hz and were extracted from the 300-Hz low-pass filtered signal. In this way, we were able to record spikes and LFPs simultaneously.

At an initial stage of the experiments, a “search procedure” was used and consisted of recording cortical activity induced by clicks, noise bursts and tone pips (from 500 Hz to 32 kHz, 1/8 octave step). This procedure provided a rough estimate of the tonotopy and the amplitude of LFPs. Moreover, electrodes were placed at a depth where the (negative) amplitude of stimulus-induced LFPs was near maximal (region of the border between layer III and IV—Szymanski et al., 2011).

#### Data analysis

**Figure 2:**
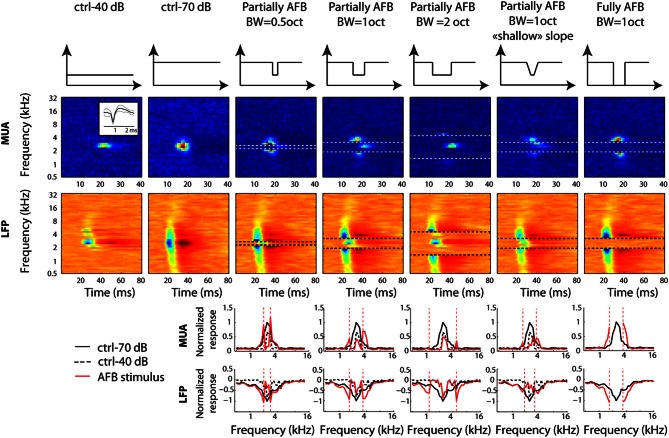
**Neural tuning of individual recordings obtained from one selected example at a given location in primary auditory cortex.** First row: long-term spectrum of all stimulus conditions. Each column corresponds to a stimulus condition. Second and third rows: STRFs derived from MUA and LFPs, respectively. Horizontal dotted lines represent the edge frequencies of the notch. Inset in the first column and second row represents the averaged waveform (±2 standard deviations) for the MUA at that particular location. Fourth and fifth rows: frequency “profile” obtained by taking the maximum firing rate of MUA, or by taking the minimal amplitude of LFPs, in the 10–40 ms time window, respectively. Vertical dotted lines represent the edge frequencies of the AFB. In order to permit direct comparison of neural responses between control and AFB conditions (red lines), the ctrl-70 (black lines) and ctrl-40 (dotted lines) conditions are added to each figure. Neural responses are greatly enhanced at the edges of the notch and decreased within the notch. The largest increase of responses is observed for the sharp contrast and the full-notch conditions (seventh column).

**Figure 3:**
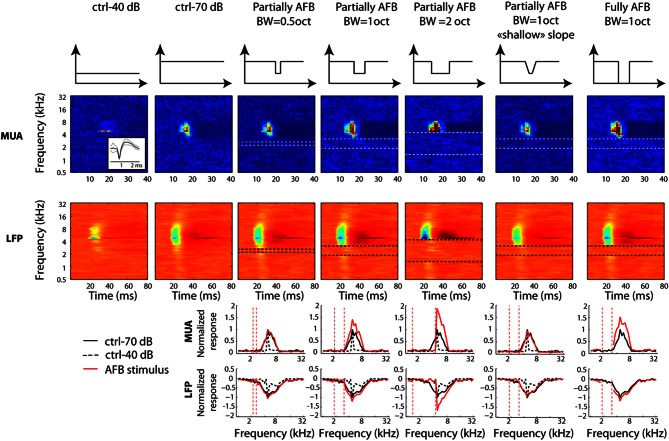
**Neural tuning of individual recordings obtained from one selected example at a given location in primary auditory cortex.** Otherwise as Figure 2. Note that the neural receptive field is unchanged when the notch is at remote frequencies.

All results were computed using custom MATLAB routines. Multi-unit activity or “spike events” were detected by using an amplitude threshold on the high-pass filtered data. The median was calculated on the negative values of the filtered signal; the threshold was then set to six times the median (see Quiroga et al., 2004 for a similar method). The spike waveforms were inspected visually throughout the experiments to ensure that they had a typical shape; inserts in Figures 2, 3 show the typical shape of multiunit activity.

The methodology for computing STRFs was similar to that used in previous studies (Valentine and Eggermont, 2004; Norena et al., 2008). Briefly, STRFs for MUA were determined by constructing poststimulus time histograms (PSTHs), with time bins of 1 ms for each tone pip frequency. In other words, spikes falling in the averaging time window (starting at the stimulus onset and lasting 100 ms) are counted. Because the average interstimulus interval in the stimulus ensemble is smaller than the averaging time window, a spike can be counted in the PSTH of several pip frequencies. STRFs for LFPs were obtained by a similar procedure, except that the LFP waveforms (0–80 ms after stimulus onset) were averaged for each appropriate tone pip frequency. The maximal MUA response (or the minimal LFP amplitude) within the 10–40 ms time window after stimulus onset and over all frequencies was obtained from the ctrl-70 STRF. All STRFs (including those obtained from the ctrl-70 condition) are then normalized by dividing the mean neural activity by this single value. This normalization was aimed at minimizing the firing rate variability across recording sites. By definition, the maximum neural activity for the ctrl-70 condition was 1 (at the best frequency), and usually lower than 1 for the ctrl-40 condition. One notes that values above 1 are sometimes observed in the AFB conditions (i.e., at the edge frequencies of the AFB); this indicates that the maximum of absolute firing rate in the AFB conditions is larger than the maximum of absolute firing rate in the ctrl-70 condition (see Figures 2, 3). This normalized mean neural activity is the dependent variable displayed in the STRFs (Figures 2, 3).

In order to compare the STRFs obtained from control and stimuli producing an AFB (and for display purpose), the differences between their frequency profiles were computed. The frequency profiles were obtained from the normalized STRFs by taking the maximum neural activity within a time window of 10–40 ms for each tone pip frequency. For the frequencies outside the AFB, which were presented at 70 dB, the responses were compared to the corresponding frequencies obtained from the ctrl-70. For the frequencies inside the AFB, which were presented at 40 dB, the responses were compared to the corresponding frequencies obtained from the ctrl-40.

Finally, the patterns of excitation, namely the neural population activity over the tonotopic axis, were obtained for the AFB conditions: for each tone pip frequency, the averaged normalized activity is derived for all MUA. Then, instead of plotting neural activity (for a given cortical site) as a function of stimulus frequency, neural activity evoked by a given frequency is plotted as a function of the best frequency of neurons (obtained from many cortical sites). The pattern of excitation could be obtained and plotted for each tone pip frequency. Assuming that auditory information is represented as a “rate-place” code in the auditory cortex, the pattern of excitation may be closer to what downstream neurons read out during stimulation. In other words, the cortex may not directly detect changes in the neurons' best frequencies but rather read out the population activity, namely neural activity along the tonotopic axis. We were particularly interested in the pattern of excitation of the edge-in and edge-out frequencies of the AFB, as we suspected that the pattern of excitation would be modified at these frequencies in the AFB conditions compared to the control conditions.

#### Statistics

Before applying any statistical test we first verified the normality of the distribution in order to validate or not the use of parametric tests. As all distributions followed the Normal law, we then used the parametric Student test (*t*-test) to compare two distributions or one distribution against zero. Significativity thresholds were adjusted according to the number of comparisons (Bonferroni's correction). First, the group analysis was carried out on sites with “significant” STRFs for the 70-ctrl condition: the maximal response of the 70-ctrl STRFs within the 10–40 ms time window had to be significantly larger than the “background activity” (computed from the neural activity over the 49 frequencies and 100 time bins, so 4900 values). As 1470 comparisons were made (30 time bins × 49 frequencies), the significativity threshold was adjusted accordingly (Bonferroni correction, *p* = 0.05/1470). Second, all other comparisons between data sets and a reference value (or data sets between ech other) were also Bonferroni corrected when needed. For instance, when the differences in firing rate between control and AFB conditions were compared to 0 for 32 different frequencies (±2 octaves on either side of the AFB center), the significance value was adjusted accordingly (*p* = 0.05/32). As the center frequency of AFB was usually centered on neuron's BF, the number of recordings was larger for sites with BF near the center frequency of the AFB than at remote frequencies. Overall, the number of recordings as a function of the distance from the center frequency of the AFB were comprised between 24 (BFs remote from AFB center) and 117 (BFs at or near the AFB center).

## Results

The aim of the present study was to investigate the cortical representation of spectral edges in auditory cortex. A total of 317 multi-unit activity (MUA) recordings were obtained from the primary auditory cortex of 10 anesthetized guinea pigs. The median for the distribution of best frequencies derived from the STRFs was 11,314 Hz (lower and upper quartiles were 7336 Hz and 20,749 Hz, respectively).

### Cortical representation of a broad-band stimulus with an attenuated frequency band (AFB)

Here, we investigated the cortical representation of the frequencies composing a multi-tone stimulus. In particular, we focused on the representation of spectral edges (edge-in and edge-out frequencies) of the AFB. As we were also interested in studying whether the representation of the edge frequencies is sensitive to their local acoustic context (the spectral shape around the edge frequencies), the width, depth and sharpness of the AFB were varied.

### Individual examples

Figure 2 depicts a representative example of MUA and LFP responses obtained for the different conditions of multi tone stimuli (the long-term frequency spectrum of the stimuli is shown in the first row). For this example, the center frequency of the AFB was chosen to correspond to the best frequency (BF) of the MUA (around 2348 Hz). When comparing the responses at the edge-out frequencies with those obtained from the 70-dB control condition, one observes a dramatic increase. Remarkably, there was a clear neural response (in terms of multi-unit activity) at the upper edge-out frequencies for the 2-octave AFB condition (column 5), even though both spectral edges fall outside of the MUA receptive field recorded in control conditions. The increase of responses at the edge-out frequencies was larger for the sharp contrast (compare columns 4 and 6) and for the large contrast (compare columns 4 and 7) conditions. On the other hand, when comparing the responses within the AFB with the 40 dB control condition, one observes a dramatic decrease of responses in all conditions, especially for edge-in frequencies.

Figure 3 shows an additional example, where the BF (4362 Hz) of MUA was almost 1 octave above the center of the AFB (2378 Hz). This example illustrates that responses are not modified when the frequency range of the AFB is far away from the MUA's receptive field (column 3). However, neural responses were broadly increased when the upper edge of the AFB was near (columns 4 and 7) or overlapped with the receptive field (column 5). Once again, this example shows that the neural enhancement is larger for the conditions with sharp (compare columns 4 and 6) and large (compare columns 4 and 7) spectral contrast.

### Group data

**Figure 4:**
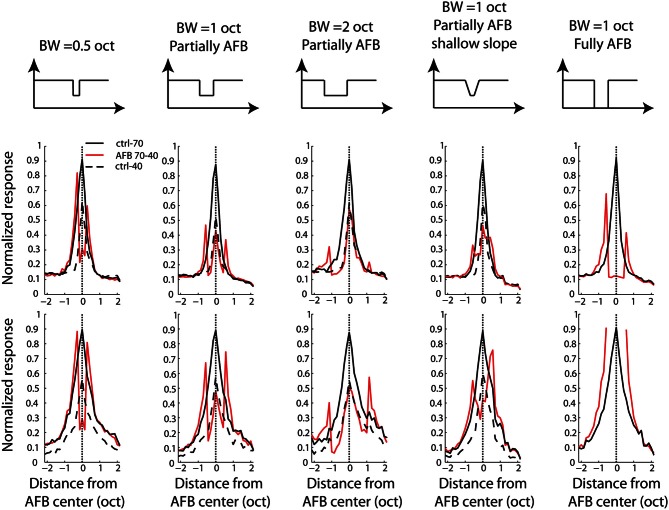
**Average frequency profile for all stimulus conditions, for one BF position relative to the AFB center (neural BF corresponding to the AFB center) for MUA (first row) and LFPs (second row).** Frequency profiles are obtained by taking the maximal normalized firing rate (for MUA, or the minimal normalized amplitude for LFPs) between 10 and 40 ms after stimulus onset. Each column corresponds to an AFB condition. Schematics illustrating the long-term spectrum of the acoustic conditions are shown at the top of the figure. First column: half-an-octave partially AFB. Second column: one-octave partially AFB. Third column: two-octaves partially AFB. Fourth column: shallow-slope partially condition. Fifth column: fully AFB. Both control conditions (at 70 dB SPL, continuous black line and at 40 dB SPL, dashed black line) are shown in all panels to permit a direct comparison between control and AFB conditions (red line). Neural responses were greatly enhanced at both edges of the notch, and reduced within the notch. The neural enhancement at both edges of the AFB was sensitive to the sharpness and the depth of the spectral contrast (compare 2nd and 4th columns, and 2nd and 5th columns, respectively).

The frequency profiles averaged over the recordings where the BF corresponded to the center of the AFB are shown in Figure 4. On average, the cortical responses are greatly enhanced at both the upper and lower edge-out frequencies, and decreased within the frequency range of the AFB. The enhancement of responses at both upper and lower edge-out frequencies was maximal for the sharp and the large contrast conditions.

**Figure 5:**
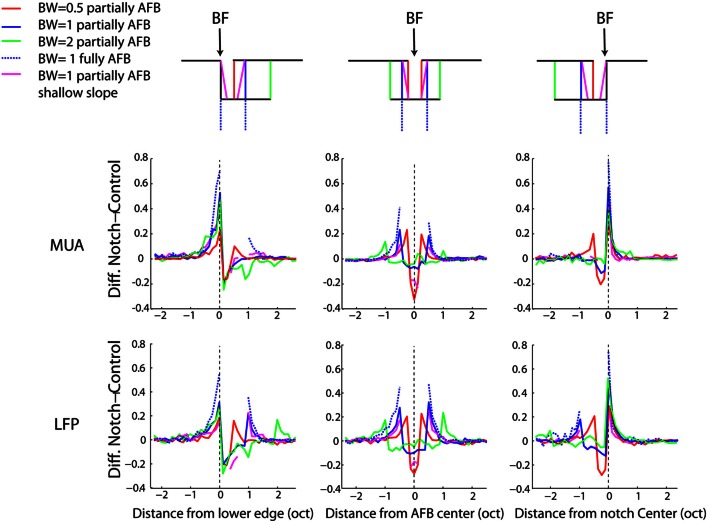
**Average difference between the AFB conditions and the control conditions for three positions of the AFB relative to the neural BF, as a function of frequency.** Each column represents a position of the AFB relative to the neural BF. Schematics illustrating all stimulus conditions and the location of BF relative to the AFB are shown at the top of the figure. The first and second rows represent the average MUA and LFP, respectively. First column: average data for neurons with BF corresponding to the lower edge of the AFB. Second column: average data for neurons with BF corresponding to the AFB center. Third column: average data for neurons with BF corresponding to the upper edge of the AFB. Neural responses at both edges of the notch are largely enhanced, while they are reduced within the notch. The enhancement of neural responses was maximal for the fully AFB and minimal for the half-an-octave condition. The reduction of responses within the AFB was larger when the BF corresponded to the lower edge of the AFB (vs. upper edge).

We next computed the difference between the frequency profiles of neural responses obtained from the AFB stimuli and those obtained from the control stimuli (see methods). This comparison was carried out for three specific positions of BF relative to the center of the AFB: when BF corresponded to the lower edge (1/8 octave), the upper edge (±1/8 octave) and the center of the AFB (±1/8 octave). The average differences between the frequency profile of AFB and control stimuli for three positions of BF relative to the center of the AFB are shown in Figure 5. The effects of the AFB stimuli relative to the control stimuli were tested statistically for both MUA and LFPs. As the results were generally not different between MUA and LFPs, we did not discriminate between these two signals in the rest of the manuscript. In other words, when a statistical difference is reported, this applies for both MUA and LFPs.

The neural enhancement for the edge-out frequencies was statistically significant for all widths of AFB and for the three positions of BF relative to the center of the AFB (*p* < 0.0014). It is worth mentioning that cortical responses were increased by about 70% for the fully AFB (when BF corresponded to either lower or upper spectral edge). Moreover, the neural enhancement for the edge-out frequencies was sensitive to the sharpness and the depth of the contrast. Indeed, cortical responses at edge-out frequencies were significantly larger for the sharp contrast condition (vs. the shallow-slope condition, *p* < 0.05) and for the 1 octave large contrast condition (vs. the 1 octave moderate contrast condition, *p* < 0.05), for all 3 positions of BF. The enhancement at the edge-out frequencies was also sensitive to the width of the AFB; indeed, the enhancement was smaller for the 0.5 octave condition compared to the 1 and 2 octave conditions (*p* < 0.05, 1 and 2 octaves conditions were not different from each other).

Besides the neural enhancement at the edge-out frequencies, there was a significant suppression of responses at the edge-in frequencies for the three positions of BF and for all notch widths (*p* < 0.0014, except for the 2-octave condition, and when BF was positioned at the upper edge frequency of the AFB). Interestingly, one notes that this neural suppression at edge-in frequencies was asymmetric for the 2-octave condition: the suppression was stronger when BF corresponded to the lower edge of the AFB (versus when BF corresponded to the upper edge of the AFB) (*p* < 0.05). Finally, the suppression at frequencies around the center of the AFB was largest for the 0.5 condition, when the BF corresponded to the center of the AFB.

**Figure 6:**
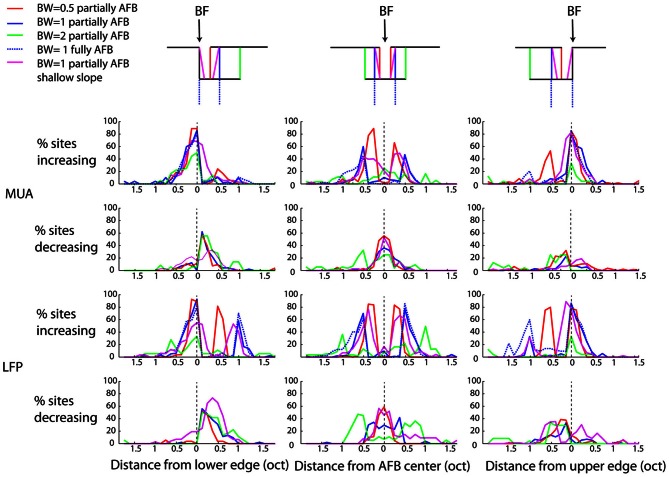
**Percentage of sites showing an increased response (first and third rows, for MUA and LFPs, respectively), or a decreased response (second and fourth rows, for MUA and LFPs, respectively).** Otherwise as Figure 4. Nearly 90% of sites showed an increased response at both edges of the AFB. The percentage of sites showing decreased responses within the notch is more variable and asymetric: 60% of sites were decreased when the BF corresponded to the lower of the notch, while 40% of sites were decreased when the BF corresponded to the upper edge of the notch.

While we found, on average, a clear (and significant) effect of the AFB on neural responses at the edge-in (suppression) and edge-out (enhancement) frequencies (Figures 4, 5), the prevalence of these changes, namely whether they concern a majority of recording sites or not, is unknown. The percentages of recording sites showing at least 20% increase or decrease as a function of frequency for the three positions of BF relative to the center of the AHL are shown in Figure 6. Nearly 90% of the recording sites showed an increase of neural responses at the lower and upper edge-out frequencies, while a decrease of neural responses at edge-in frequencies was observed in around 60% of the recordings. This suggests that the cortical changes induced by the notched stimuli are very systematic. It is also worthwhile to mention that while the percentages of sites showing an increase at the edge-out frequencies is similar whether the BF corresponded to the lower edge or the upper edge of the AFB, the percentages of sites showing a decrease at the edge-in frequencies is larger when BF corresponded to the lower edge of the AFB (around 60% of the sites) than when BF corresponded to the upper edge of the AFB (around 20–30% of the sites). This result is consistent with the asymmetry in suppression observed from the averaged data (Figure 5), showing that neural suppression of edge-in frequencies is stronger at the lower edge of the AFB than at the upper edge (Figure 6) (see discussion for putative functional implications).

### Population activity over the tonotopic axis

**Figure 7:**
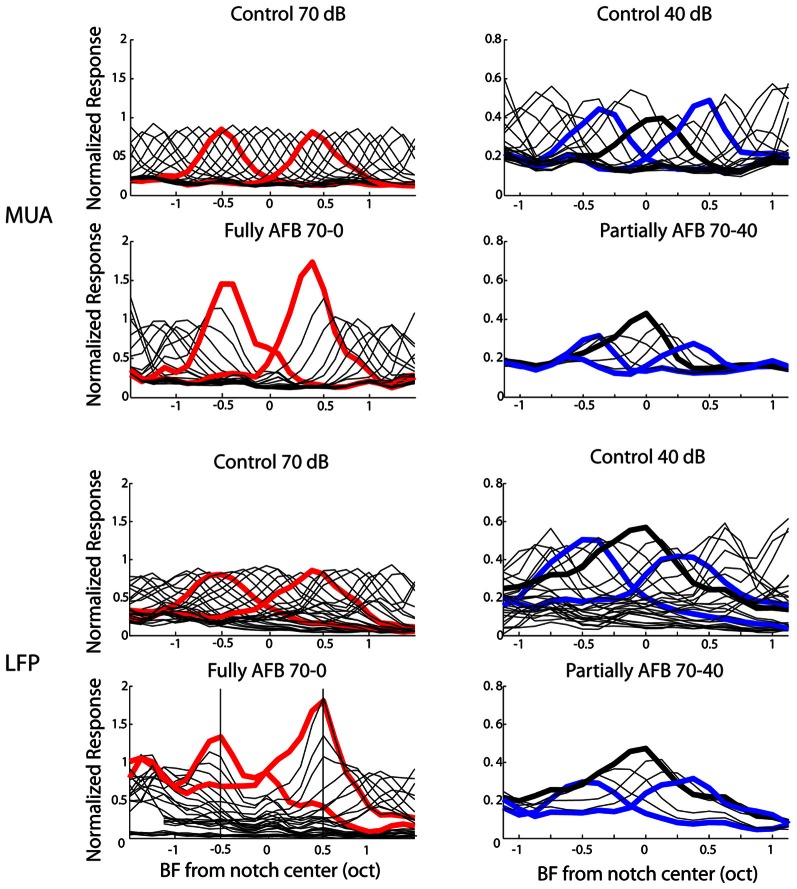
**Averaged “patterns of excitation” for MUA (first and second rows) and LFPs (third and fourth rows) in the 1-octave AFB condition.** Neural activity was normalized to the maximal activity obtained in the ctrl-70 condition and was smoothed with a moving average. In the first (for MUA) and third (for LFPs) rows, excitation patterns for the ctrl-70 (first column) and the ctrl-40 (second column) are represented. In the first column, the second (for MUA) and fourth (for LFPs) rows represent the excitation patterns for the fully AFB condition. The “excitation patterns” for frequencies at the upper and lower edge-out frequencies are represented by thick red lines. In the second column, the second (for MUA) and fourth (for LFPs) rows represent the excitation patterns for the partially AFB condition. The excitation patterns for frequencies at the upper and lower edge-in frequencies are represented by thick blue lines (excitation pattern at center frequency of the AFB is represented by thick black line). This figure shows that the representations of frequencies at both edge-out frequencies of the AFB as well as at edge-in frequencies are greatly “distorted”.

Thus far, neural data were analyzed with an emphasis on the characteristics of neural tuning. From a neural decoding point of view, on the other hand, a more relevant representation may be the spatio-temporal distribution of population activity. The cortex processes this dynamic and distributed population activity in real time over remote cortical regions. In order to provide a representation of neural activity closer to what may be relevant in the auditory cortex, we derived an excitation pattern (thought to approximate population activity) from MUA and LFP recordings. One notes that this representation was made possible by our matrix electrodes which allowed a relatively dense sampling of cortical responses over the tonotopic axis. The excitation patterns were obtained for each tone pip frequency presented in the multi-tone stimuli. Instead of representing the individual or average activity of cortical neurons as a function of frequency, the average neural activity was represented as a function of neural BF for each given stimulus frequency. This representation gives an estimate of the spatial representation (or population activity) of each tone pip frequency over the tonotopic axis (Figure 7). The resulting excitation patterns in the control condition (stimulus with a flat spectrum) were very homogeneous over frequency and resembled a Gaussian-shaped curve: for a given tone pip frequency, the activity is maximal for neurons with BF corresponding to that frequency (by definition), while neural activity decreases gradually for neurons whose BF is further from that frequency. More interestingly, the excitation patterns obtained from AFB stimuli were very different from those derived from control conditions. At the edge-out frequencies, the excitation patterns were not only increased (in terms of neural response amplitude, as already shown above) but they became broader. On the other hand, at the edge-in frequencies, the excitation patterns were decreased in amplitude and became narrower.

**Figure 8:**
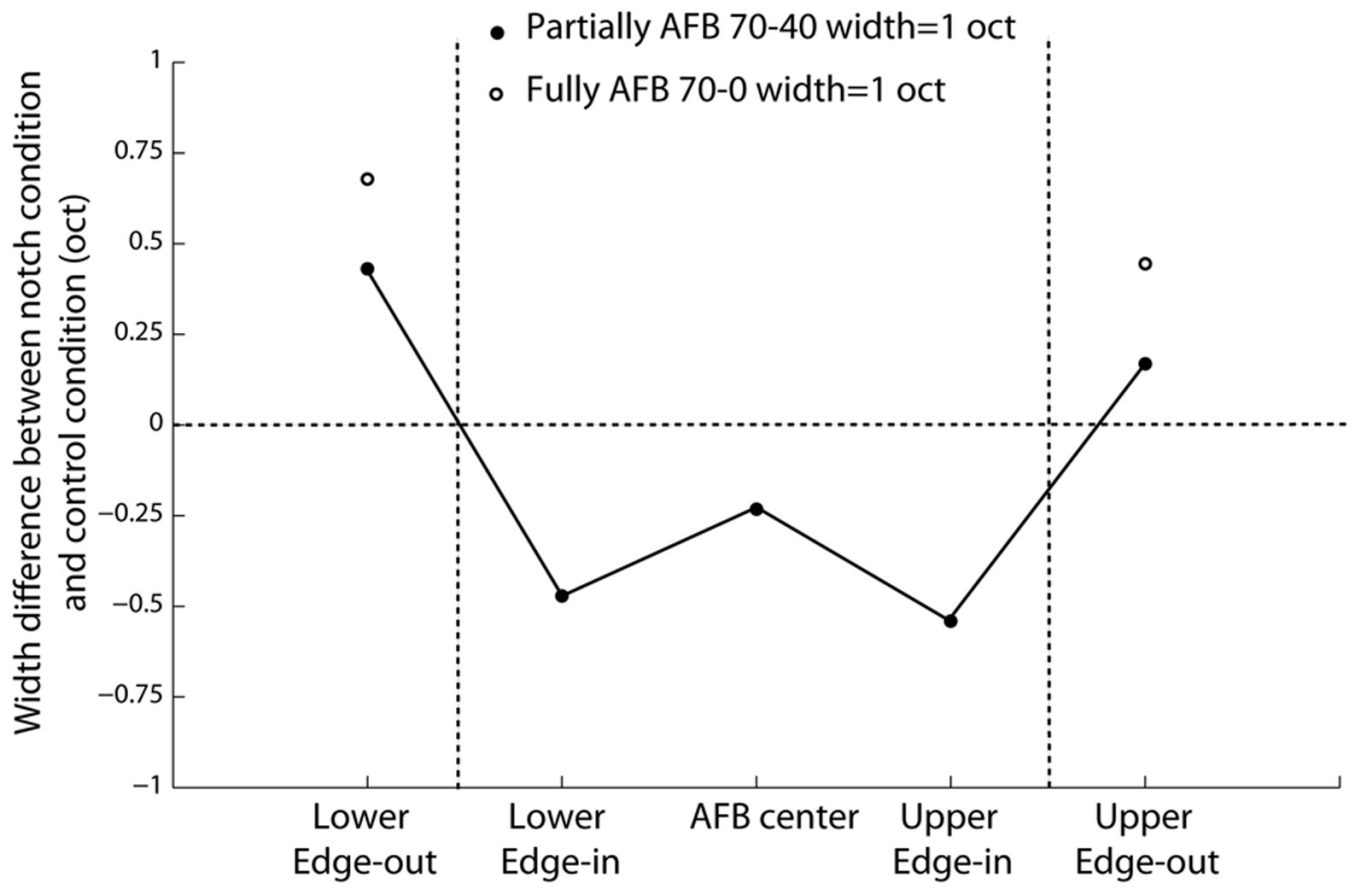
**Difference (in octaves) in the width of the “excitation patterns” between the two AFB conditions (one-octave and fully AFB) and the control conditions (40-dB within the AFB and 70-dB outside the AFB).** Positive values indicate increased width, while negative values indicate decreased width. The width of the “excitation patterns” gives an estimate of the number of neurons involved in the representation of a given frequency.

In order to investigate whether the AFB stimuli modified the cortical representation of edge-in and edge-out frequencies, the width of the excitation patterns was derived (at the normalized neural activity of 0.2). The respective widths obtained from control and AFB stimuli were then compared statistically (Figure 8). During the stimulation with the AFB stimuli, the representation of the edge-out frequencies was expanded (*p* < 0.05), while the representation of the edge-in frequencies was narrowed (*p* < 0.05). These results suggest that the cortical representation of stimulus frequencies (in terms of the amplitude of the response and number of the neurons involved) is highly dynamic and depends heavily on the overall acoustic spectrum or acoustic context.

### Cortical representation of a broad-band stimulus with an enhanced frequency (EF)

In order to gain further insight into the properties of the firing rate reduction on either side of a spectral edge (later called “lateral suppression”), in particular its width and asymmetry, an additional experiment was carried out. In this experiment, cortical responses were obtained from a multi-tone stimulus where all pure tones were presented at 40 dB SPL, except at one frequency which was presented at 70 dB SPL; that frequency was referred as the enhanced frequency (EF). The main purpose of this experiment was to investigate the cortical representation of tone pip frequencies surrounding the EF. Indeed, if a central mechanism exists that sharpens the neural representation of spectral edges, then one expects a decrease of neural responses at frequencies adjacent to the EF, as this would produce an increase of the peak-to-valley ratio.

**Figure 9:**
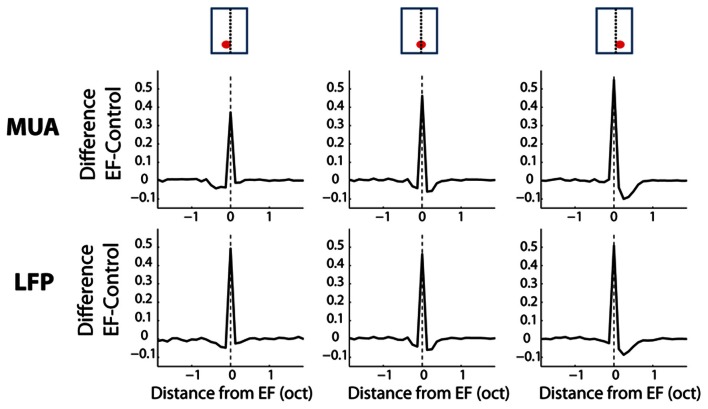
**Average difference between the stimulus condition with an EF and the control conditions for three positions of the EF relative to the neural BF, as a function of frequency.** Each column represents a position of the EF relative to the neural BF. At the top of the figure, schematics illustrate all stimulus conditions, namely the location of the receptive field relative to the EF (see text for more details). First column: average data for neurons with BF lower than the EF. Second column: average data for neurons with BF corresponding to the EF. Third column: average data for neurons with BF higher than the peak frequency. One notes the neural suppression at frequencies adjacent to the peak frequency and the asymmetry of this neural suppression (suppression is stronger from low to high frequencies).

The average differences between the frequency profiles of the EF stimulus and control stimuli for three positions of BF relative to the EF are shown in Figure 9. Neural responses obtained from the EF stimulus at the EF were compared to the ctrl-70 and those at other frequencies were compared to the ctrl-40. Cortical responses at the EF were largely increased in the EF condition compared to those in the control condition (*p* < 0.0014). On the other hand, neural responses were significantly decreased on either side of the EF (up to ¼ octave away from the EF, *p* < 0.0014—condition where the EF corresponded to BF). Interestingly, this decrease was largely asymmetric over frequency: the decrement of cortical responses was stronger (and slightly wider) towards high frequencies than towards low frequencies. In the condition where BF was lower than the EF, only one frequency below the EF was significantly suppressed (*p* < 0.0014). In the condition where BF was higher than the EF, frequencies up to 3/8 octave above the EF were suppressed (*p* < 0.0014). The width and the asymmetry of the suppressed sidebands observed in this stimulus condition are broadly consistent with the neural changes produced by the AFB stimuli (Figures 5, 6) (see discussion).

## Discussion

The present study was aimed at investigating whether there is an enhancement, in auditory cortex, of the representation of spectral edges in acoustic stimuli. Overall, we show that the cortical representation of the acoustic spectrum tends to enhance the spectral edges. As the stimuli used in this study have spectral contrasts or edges only when they are time-averaged over a few hundreds of milliseconds, our results imply that auditory centers integrate the stimulus spectrum over hundreds of milliseconds. More specifically, in the condition where a frequency band was attenuated, we observed that cortical responses were increased near the edge-out frequencies, whereas they were reduced for the edge-in frequencies. Interestingly, by estimating the neural population activity over the tonotopic axis, we also found that the cortical response profile following the presentation of a stimulus with an AFB was greatly altered: the relative number of sites responsive to the edge-out frequencies was increased, while the relative number of sites responsive to the edge-in frequencies was decreased (compared to the number of sites representing the frequencies remote from the AFB). These cortical changes were sensitive to the properties of the AFB, namely its width, depth and sharpness. These changes were highly systematic, being present in the majority of cortical recording sites. In the condition where the sound level of a single tone frequency was increased, neural activity was reduced at the neighboring frequencies of the enhanced frequency.

### The spectro-temporal integration investigated by other studies

The effects “at a distance” between frequencies presented in a given temporal sequence reported in the present study are reminiscent of those reported previously. In particular, presenting a pulsated tone pip at a given frequency for seconds to minutes has been shown to produce a decrement of cortical responses not only at the tone pip frequency but also at nearby frequencies (Condon and Weinberger, 1991; Ulanovsky et al., 2004). The spectro-temporal interactions of acoustic stimuli in auditory cortex have been investigated using two-tone sequences (Shamma, 1985; Shamma and Symmes, 1985; Calford and Semple, 1995; Rajan, 1998). A complex pattern of firing suppression and facilitation has been reported, which depends on the frequency separation and the delay between the two tones (Brosch and Schreiner, 1997, 2000; Brosch and Scheich, 2008; Sadagopan and Wang, 2010). One notes that little difference has been found between these effects in multi-unit and single unit activity (Brosch and Schreiner, 1997, 2000; Brosch et al., 1999). Our demonstration that cortical responses (multi-unit activity and local field potentials) are either suppressed or enhanced depending on the stimulus context is consistent with these studies. However, the above studies did not specifically address the cortical representation of spectral edges embedded in spectrally complex acoustic stimuli or the sensitivity of this representation to the characteristics of the spectral edges (width, depth, sharpness). Indeed, while neural enhancement at spectral edges has been predicted by various computational models (Shamma, 1985; Yost, 1986; Gerken, 1996; Parra and Pearlmutter, 2007), there is to our knowledge only one experimental study showing cortical enhancement near the cutoff frequency of 2-octave wide multi-tone stimuli (Gourévitch et al., 2009). The present study, however, extends the latter by reporting, for the first time, the effects of the physical characteristics of the spectral contrast (sharpness, depth and width) and by documenting neural responses for frequencies nearby and within spectral notches. Our study also provides new information about the width and asymmetry of lateral suppression that are crucial for computational models and the functional implications of these mechanisms (see below). Finally, while we did not investigate specifically the effects of rate and spectral density of our acoustic stimuli on cortical responses (Blake and Merzenich, 2002; Valentine and Eggermont, 2004; Norena et al., 2008), it is likely that the central changes reported in the present study are sensitive to these parameters. Presentation rate and spectral density have to be high enough to fall within the spectro-temporal integration constants of cortical neurons. Indeed, very small presentation rate should not produce any edge enhancement, even for spectrally dense stimuli, and reversely.

### Mechanisms of neural enhancements and decrements at the spectral edges

The malleability of cortical responses reported in the present study are produced by acoustic stimuli presented passively for only 3 min, in contrast to studies that reported rapid modifications in frequency tuning but during/after active listening (Edeline et al., 1993; Fritz et al., 2003, 2005; Elhilali et al., 2007) or after prolonged (on the order of weeks) stimulation (Norena et al., 2006; Kim and Bao, 2009). The rapidity of these cortical changes precludes the involvement of slow cortical changes such as those involved in homeostatic plasticity (Watt and Desai, 2010) or long-term depression and potentiation (Buonomano and Merzenich, 1998). Instead, they are likely the results of one or a combination of relatively fast mechanisms, occurring on the order of milliseconds to seconds or minutes.

The first (fast) mechanism that comes to mind to account for our results is lateral inhibition. Indeed, it has long been recognized that lateral inhibition could be used by sensory systems to sharpen/enhance the representation of stimulus contrasts (Hartline et al., 1956; Ratliff and Hartline, 1959; Von Bekesy, 1967, 1969a,b; Marr and Hildreth, 1980). The presence of lateral inhibition has been suspected at virtually all levels of the central auditory system using various methodology such as whole-cell recordings (Wu et al., 2008), electrophysiology (two-tone sequences, effects of hearing loss) (Shamma and Symmes, 1985; Calford et al., 1993; Rhode and Greenberg, 1994; Calford and Semple, 1995; Suga et al., 1997; Rajan, 1998, 2001; Wang et al., 2002; Noreña et al., 2003) and pharmacology (Yang et al., 1992; LeBeau et al., 2001). Lateral inhibition is likely to contribute to our results when tones of different frequencies overlap in time. Moreover, tones are presented in random temporal sequences with a relatively short average inter-stimulus interval (500 ms for one frequency, or nearly 60 ms for one-octave frequency band—roughly the width of the STRFs) suggesting that the cortical activity induced by a tone at a given time also depends on the tones presented shortly before it (Brosch and Schreiner, 1997, 2000; Brosch and Scheich, 2008; Sadagopan and Wang, 2010). These post-stimulatory effects on neural activity have been shown to result from synaptic inhibition up to 100 ms after stimulus presentation (Wehr and Zador, 2005). At longer delays, on the other hand, other mechanisms involved in synaptic depression, such as receptor desensitization, vesicle depletion and changes in presynaptic release probability are thought to be at work.

In summary, we propose that the cortical changes reported in the present study are likely the results of different mechanisms such as synaptic inhibition and synaptic depression. Reduced synaptic inhibition and/or synaptic depression produced by frequencies falling in the AFB could result in a relative increase of responses at the edge-out frequencies, while the enhanced synaptic inhibition and/or synaptic depression produced by the (enhanced) edge-out frequencies could in turn reduce the responses at the edge-in frequencies. One further important question relative to the mechanisms enhancing spectral contrasts is whether they operate at the cortical level or are inherited from earlier stages of the auditory pathway. The very similar pattern of responses for MUA and LFPs (the latter known to represent mainly the thalamic inputs sent toward the cortex) (Mitzdorf, 1985; Steinschneider et al., 1992) suggests that the enhancement of spectral contrasts observed in cortex is largely inherited from lower levels. Consistent with a sub-cortical contribution to the cortical changes reported in the present study, a complex pattern of firing suppression has been evidenced in the cochlear nucleus (Rhode and Greenberg, 1994). Further studies will be needed to investigate this important question. Finally, the present study has been carried out on anesthetized animals (mixture of ketamine and xylazine); consequently, it is unclear whether the results reported here also apply to awake animals. However, a study carried out in the primary auditory cortex of awake macaques shows that anesthetics only marginally modifies the pattern of neural suppression and facilitation produced by two-tone sequences (Brosch and Scheich, 2008). The latter study suggests that the cortical responses produced by our stimuli may also apply to awake preparation.

### Sensory input conditions mimicked by our stimuli with attenuated frequency band

The stimuli used in the present study can be thought as producing acoustic environments with different spectral profiles when time-averaged over a few hundreds of milliseconds. One can wonder whether these synthetic stimuli mimic natural sensory input conditions for the auditory system.

One pattern of sensory inputs that may be mimicked by our notched stimuli is that produced by a complex broadband environment in presence of sharp notched hearing loss. Hearing losses restricted to a given frequency band (i.e., referred to as an audiogram with notches) have been reported in many studies (Gates et al., 2000; McBride and Williams, 2001; Rabinowitz et al., 2006; Nondahl et al., 2009; Etchelecou et al., 2011). Assuming that the time-averaged acoustic background is “flat”, this particular shape of hearing loss is thought to result in an averaged pattern of (rate-place) activity in the cochlear nerve with a dip corresponding to the hearing loss region. More specifically, frequency regions outside hearing loss are evenly stimulated, while the frequency region of hearing loss receives only weak stimulation, if any (Gerken, 1996). In other words, our notched stimuli mimic the contrast in the averaged rate-place sensory inputs over the tonotopic axis in presence of hearing loss. As the pattern of sensory inputs provided by the AFB stimuli resembles the averaged pattern of sensory inputs in presence of notched hearing loss, the AFB stimuli can be interpreted as producing an acute “functional deafferentation” or “artificial hearing loss” (Pantev et al., 1999; Norena et al., 2000; Okamoto et al., 2007). In this context, our notched stimuli can be considered as an equivalent of the stimulus used in vision to produce an “artificial scotoma,” i.e., moving lines or random dots stimulating the visual field around a small non-stimulated area (Ramachandran and Gregory, 1991; Pettet and Gilbert, 1992; Das and Gilbert, 1995; DeAngelis et al., 1995). One notes, however, that our stimuli do not model some typical characteristics accompanying cochlear damage, such as the decrease in spontaneous activity in the cochlear nerve within the frequency range of cochlear damage (Liberman and Dodds, 1984), the neural degeneration of cochlear fibers (Kujawa and Liberman, 2006, 2009) and/or the broadening of auditory filters (Glasberg and Moore, 1986).

The results of the present study may give some insights into the sensitivity of the auditory cortex to the characteristics of acute hearing loss. These properties are potentially important for the understanding of the functional implications of the cortical changes produced by acute hearing loss, such as tinnitus, for example (Norena, 2011; Noreña and Farley, 2013). One notes that the relationship between cortical changes and the characteristics of hearing loss is relatively difficult to study in practice as (noise-induced) hearing loss is generally variable (Loeb and Smith, 1967; Atherley et al., 1968). In conclusion, our study suggests that the cortical changes produced by acute hearing loss could be sensitive to the sharpness, depth and width of hearing loss. Moreover, while the cortical changes observed in the present study are short-term, it is possible that a more prolonged exposure to the AFB stimulus could induce long-lasting changes such as those produced by chronic hearing loss or reported in previous studies (Robertson and Irvine, 1989; Rajan et al., 1993; Norena and Eggermont, 2005; Norena et al., 2006; Pienkowski et al., 2013).

### Properties of central inhibition

Our results also provide some information about the properties of lateral suppression of firing (either it is produced by synaptic inhibition and/or synaptic depression) in the central auditory system. The bandwidth of suppressed sidebands derived from this study (0.25–0.4 octave) closely approximates the lateral inhibitory networks described by Shamma (1985, 0.3 octave) and Yost (1986, 0.2 octave). We also show that lateral suppression is asymmetric as a function of frequency with a stronger and wider suppression produced toward high frequencies (suppression was significant up to around 0.4 octaves above the spectral peak) than toward low frequencies (suppression was significant up to 0.25 octaves below the spectral peak) (Figure 9). This particular pattern of asymmetric inhibition is consistent with the results of (Zhang et al., 2003) for high BF neurons. As the pattern of vibration of the basilar membrane is asymmetric (slope is shallower on the basal side of the cochlea compared to the apical side), leading to the corresponding asymmetric pattern of excitation in the cochlear nerve, it has been suggested that the asymmetry of central inhibition (stronger inhibition from low to high frequencies) may further refine the central representation of spectral edges (Suga, 1995; Okamoto et al., 2007).

We have proposed that the “Zwicker tone,” the tonal and faint illusory percept produced after the presentation of a notched noise (broadband noise containing a suppressed frequency band) (Zwicker, 1964; Lummis and Guttman, 1972; Wiegrebe et al., 1996; Franosch et al., 2003), could be interpreted as a model of transient tinnitus (Norena et al., 2000; Noreña and Eggermont, 2003; Parra and Pearlmutter, 2007). The “Zwicker tone” can also be induced by low-pass or high-pass noises, although the former is more efficient to produce the sensation (Lummis and Guttman, 1972). It is interesting to note that this asymmetry for producing the “Zwicker tone” might be related to the asymmetry in neural suppression reported in the present study (larger neural suppression at lower edge-in frequencies vs. upper edge-in frequencies).

### Implications for neural coding

The present study shows that the cortical representation of spectral edges is enhanced (more neurons are dedicated to the representation of spectral edges). A putative link between stimulus importance and its representational size in the primary auditory cortex has been suggested (Rutkowski and Weinberger, 2005). Our study further suggests that the representational size of spectral cues may be dynamically enhanced in cortex. This may improve the processing of relevant spectral cues (edges) within the ever changing acoustic environment.

It has been suggested that the responsiveness (gain) of sub-cortical and cortical neurons could be dynamically adapted to the statistics (mean and variance) of stimuli. This mechanism provides an elegant solution to the dynamic range problem (Viemeister, 1988) by adjusting the input–output function of neurons to the distribution of input levels (Dean et al., 2005, 2008; Watkins and Barbour, 2008, 2011; Rabinowitz et al., 2011, 2012). These studies varied the mean and variance of stimulus level across conditions but the mean stimulus level was fixed (for single pure tone) or uniform (for noise bursts or multi-tone pips) over frequency for a given condition. Our study can be considered as an extension of these studies as the mean level was varied over frequency (mean level was low in the AFB, and high elsewhere). While the hypothesis of gain control predicts a decrease of gain for high contrast stimuli (and the reverse at low contrast stimuli—neurons become more sensitive to small level variations), our results suggest the opposite: the firing rate difference between edge-out and edge-in frequencies are maximally enhanced for sharp and deep contrast. These results emphasize the importance of considering the effects of the spectral dimension (spectral envelope) in future studies investigating contrast gain control.

### Conflict of interest statement

The authors declare that the research was conducted in the absence of any commercial or financial relationships that could be construed as a potential conflict of interest.

## References

[B1] Assmann P. F. Nearey T. M. (1987). Perception of front vowels: the role of harmonics in the first formant region. J. Acoust. Soc. Am. 81, 520–534 10.1121/1.394918 3558970

[B2] Assmann P. Summerfield Q. (2004). The Perception of Speech Under Adverse Conditions, in Speech Processing in the Auditory System (New York: Springer-Verlag), 231–308 Available at: http://link.springer.com/chapter/10.1007/0-387-21575-1_5 [Accessed July 27, 2012]. 10.1007/0-387-21575-1_5

[B3] Atherley G. R. Hempstock T. I. Noble W. G. (1968). Study of tinnitus induced temporarily by noise. J. Acoust. Soc. Am. 44, 1503–1506 10.1121/1.1911288 5702023

[B4] Baer T. Moore B. C. Gatehouse S. (1993). Spectral contrast enhancement of speech in noise for listeners with sensorineural hearing impairment: effects on intelligibility, quality, and response times. J. Rehabil. Res. Dev. 30, 49–72 8263829

[B4a] Bilsen F. A. (1977). Pitch of noise signals: evidence for a “central spectrum.” J. Acoust. Soc. Am. 61, 150–161 10.1121/1.381276 833366

[B5] Blackburn C. C. Sachs M. B. (1990). The representations of the steady-state vowel sound /e/ in the discharge patterns of cat anteroventral cochlear nucleus neurons. J. Neurophysiol. 63, 1191–1212 2358869 10.1152/jn.1990.63.5.1191

[B6] Blake D. T. Merzenich M. M. (2002). Changes of AI receptive fields with sound density. J. Neurophysiol. 88, 3409–3420 10.1152/jn.00233.2002 12466457

[B7] Bregman A. S. (1990). Auditory scene analysis: the perceptual organization of sound. Available at: http://mitpress.mit.edu/catalog/item/default.asp?ttype=2&tid=9065 [Accessed July 27, 2012].

[B8] Brosch M. Scheich H. (2008). Tone-sequence analysis in the auditory cortex of awake macaque monkeys. Exp. Brain Res. 184, 349–361 10.1007/s00221-007-1109-7 17851656

[B9] Brosch M. Schreiner C. E. (1997). Time course of forward masking tuning curves in cat primary auditory cortex. J. Neurophysiol. 77, 923–943 9065859 10.1152/jn.1997.77.2.923

[B10] Brosch M. Schreiner C. E. (2000). Sequence sensitivity of neurons in cat primary auditory cortex. Cereb. Cortex 10, 1155–1167 10.1093/cercor/10.12.1155 11073865

[B11] Brosch M. Schulz A. Scheich H. (1999). Processing of sound sequences in macaque auditory cortex: response enhancement. J. Neurophysiol. 82, 1542–1559 10.1093/cercor/10.12.1155 10482768

[B12] Buonomano D. V. Merzenich M. M. (1998). Cortical plasticity: from synapses to maps. Annu. Rev. Neurosci. 21, 149–186 10.1146/annurev.neuro.21.1.149 9530495

[B13] Calford M. B. Rajan R. Irvine D. R. (1993). Rapid changes in the frequency tuning of neurons in cat auditory cortex resulting from pure-tone-induced temporary threshold shift. Neuroscience 55, 953–964 10.1016/0306-4522(93)90310-C 8232905

[B14] Calford M. B. Semple M. N. (1995). Monaural inhibition in cat auditory cortex. J. Neurophysiol. 73, 1876–1891 7623087 10.1152/jn.1995.73.5.1876

[B15] Carlile S. Martin R. McAnally K. (2005). Spectral information in sound localization. Int. Rev. Neurobiol. 70, 399–434 10.1016/S0074-7742(05)70012-X 16472641

[B16] Carterette E. C. Friedman M. P. Lovell J. D. (1969). Mach bands in hearing. J. Acoust. Soc. Am. 45, 986–998 10.1121/1.1911575 5791617

[B17] Condon C. D. Weinberger N. M. (1991). Habituation produces frequency-specific plasticity of receptive fields in the auditory cortex. Behav. Neurosci. 105, 416–430 10.1037/0735-7044.105.3.416 1863363

[B18] Conley R. A. Keilson S. E. (1995). Rate representation and discriminability of second formant frequencies for /epsilon/-like steady-state vowels in cat auditory nerve. J. Acoust. Soc. Am. 98, 3223–3234 10.1121/1.413812 8550947

[B19a] Dai H. (2000). On the relative influence of individual harmonics on pitch judgment. J. Acoust. Soc. Am. 107, 953–959 10.1121/1.428276 10687704

[B19] Darwin C. J. (1984). Perceiving vowels in the presence of another sound: constraints on formant perception. J. Acoust. Soc. Am. 76, 1636–1647 10.1121/1.391610 6520301

[B20] Darwin C. J. (1997). Auditory grouping. Trends Cogn. Sci. (Regul. Ed.) 1, 327–333 10.1016/S1364-6613(97)01097-8 21223942

[B21] Das A. Gilbert C. D. (1995). Receptive field expansion in adult visual cortex is linked to dynamic changes in strength of cortical connections. J. Neurophysiol. 74, 779–792 7472382 10.1152/jn.1995.74.2.779

[B22] Dean I. Harper N. S. McAlpine D. (2005). Neural population coding of sound level adapts to stimulus statistics. Nat. Neurosci. 8, 1684–1689 10.1038/nn1541 16286934

[B23] Dean I. Robinson B. L. Harper N. S. McAlpine D. (2008). Rapid neural adaptation to sound level statistics. J. Neurosci. 28, 6430–6438 10.1523/JNEUROSCI.0470-08.2008 18562614 PMC6670892

[B24] DeAngelis G. C. Anzai A. Ohzawa I. Freeman R. D. (1995). Receptive field structure in the visual cortex: does selective stimulation induce plasticity? Proc. Natl. Acad. Sci. U.S.A. 92, 9682–9686 10.1073/pnas.92.21.9682 7568197 PMC40866

[B25] deCharms R. C. Blake D. T. Merzenich M. M. (1998). Optimizing sound features for cortical neurons. Science 280, 1439–1443 10.1126/science.280.5368.1439 9603734

[B26] Edeline J. M. Pham P. Weinberger N. M. (1993). Rapid development of learning-induced receptive field plasticity in the auditory cortex. Behav. Neurosci. 107, 539–551 10.1037/0735-7044.107.4.539 8397859

[B27] Elhilali M. Fritz J. B. Chi T.-S. Shamma S. A. (2007). Auditory cortical receptive fields: stable entities with plastic abilities. J. Neurosci. 27, 10372–10382 10.1523/JNEUROSCI.1462-07.2007 17898209 PMC6673154

[B28] Etchelecou M.-C. Coulet O. Derkenne R. Tomasi M. Noreña A. J. (2011). Temporary off-frequency listening after noise trauma. Hear. Res. 282, 81–91 10.1016/j.heares.2011.09.006 21986211

[B29] Franosch J.-M. P. Kempter R. Fastl H. Van Hemmen J. L. (2003). Zwicker tone illusion and noise reduction in the auditory system. Phys. Rev. Lett. 90, 178103 10.1103/PhysRevLett.90.178103 12786108

[B30] Fritz J. Elhilali M. Shamma S. (2005). Active listening: task-dependent plasticity of spectrotemporal receptive fields in primary auditory cortex. Hear. Res. 206, 159–176 10.1016/j.heares.2005.01.015 16081006

[B31] Fritz J. Shamma S. Elhilali M. Klein D. (2003). Rapid task-related plasticity of spectrotemporal receptive fields in primary auditory cortex. Nat. Neurosci. 6, 1216–1223 10.1038/nn1141 14583754

[B32] Gates G. A. Schmid P. Kujawa S. G. Nam B. D'Agostino R. (2000). Longitudinal threshold changes in older men with audiometric notches. Hear. Res. 141, 220–228 10.1016/S0378-5955(99)00223-3 10713509

[B33] Gerken G. M. (1996). Central tinnitus and lateral inhibition: an auditory brainstem model. Hear. Res 97, 75–83 10.1016/S0378-5955(96)80009-8 8844188

[B34] Glasberg B. R. Moore B. C. (1986). Auditory filter shapes in subjects with unilateral and bilateral cochlear impairments. J. Acoust. Soc. Am. 79, 1020–1033 10.1121/1.393374 3700857

[B35] Glasberg B. R. Moore B. C. (2000). Frequency selectivity as a function of level and frequency measured with uniformly exciting notched noise. J. Acoust. Soc. Am. 108, 2318–2328 10.1121/1.1315291 11108372

[B36] Gourévitch B. Noreña A. Shaw G. Eggermont J. J. (2009). Spectrotemporal receptive fields in anesthetized cat primary auditory cortex are context dependent. Cereb. Cortex 19, 1448–1461 10.1093/cercor/bhn184 18854580

[B37] Griffiths T. D. Warren J. D. (2004). What is an auditory object? Nat. Rev. Neurosci. 5, 887–892 10.1038/nrn1538 15496866

[B38] Grothe B. Pecka M. McAlpine D. (2010). Mechanisms of sound localization in mammals. Physiol. Rev. 90, 983–1012 10.1152/physrev.00026.2009 20664077

[B39] Hartline H. K. Wagner H. G. Ratliff F. (1956). Inhibition in the eye of Limulus. J. Gen. Physiol. 39, 651–673 10.1085/jgp.39.5.651 13319654 PMC2147566

[B40] Henry B. A. Turner C. W. Behrens A. (2005). Spectral peak resolution and speech recognition in quiet: normal hearing, hearing impaired, and cochlear implant listeners. J. Acoust. Soc. Am. 118, 1111–1121 10.1121/1.1944567 16158665

[B41] Houtgast T. (1972). Psychophysical evidence for lateral inhibition in hearing. J. Acoust. Soc. Am. 51, 1885–1894 10.1121/1.1913048 4339849

[B42] Kim H. Bao S. (2009). Selective increase in representations of sounds repeated at an ethological rate. J. Neurosci. 29, 5163–5169 10.1523/JNEUROSCI.0365-09.2009 19386912 PMC2717947

[B43] Kujawa S. G. Liberman M. C. (2006). Acceleration of age-related hearing loss by early noise exposure: evidence of a misspent youth. J. Neurosci 26, 2115–2123 10.1523/JNEUROSCI.4985-05.2006 16481444 PMC1855187

[B44] Kujawa S. G. Liberman M. C. (2009). Adding insult to injury: cochlear nerve degeneration after “temporary” noise-induced hearing loss. J. Neurosci. 29, 14077–14085 10.1523/JNEUROSCI.2845-09.2009 19906956 PMC2812055

[B45] LeBeau F. E. Malmierca M. S. Rees A. (2001). Iontophoresis *in vivo* demonstrates a key role for GABA(A) and glycinergic inhibition in shaping frequency response areas in the inferior colliculus of guinea pig. J. Neurosci. 21, 7303–7312 11549740 10.1523/JNEUROSCI.21-18-07303.2001 PMC6762982

[B46] Liberman M. C. Dodds L. W. (1984). Single-neuron labeling and chronic cochlear pathology. II. Stereocilia damage and alterations of spontaneous discharge rates. Hear. Res. 16, 43–53 10.1016/0378-5955(84)90024-8 6511672

[B47] Loeb M. Smith R. P. (1967). Relation of induced tinnitus to physical characteristics of the inducing stimuli. J. Acoust. Soc. Am. 42, 453–455 10.1121/1.1910600 6075938

[B48] Lummis R. C. Guttman N. (1972). Exploratory studies of Zwicker's “negative afterimage” in hearing. J. Acoust. Soc. Am. 51, 1930–1944 10.1121/1.1913052 5045252

[B49] Marr D. Hildreth E. (1980). Theory of edge detection. Proc. R. Soc. Lond., B, Biol. Sci. 207, 187–217 10.1098/rspb.1980.0020 6102765

[B50] May B. J. Prell G. S. Sachs M. B. (1998). Vowel representations in the ventral cochlear nucleus of the cat: effects of level, background noise, and behavioral state. J. Neurophysiol. 79, 1755–1767 10.1121/1.416293 9535945

[B51] McBride D. Williams S. (2001). Audiometric notch as a sign of noise induced hearing loss. Occup. Environ. Med. 58, 46–51 10.1136/oem.58.1.46 11119634 PMC1740031

[B53] Mitzdorf U. (1985). Current source-density method and application in cat cerebral cortex: investigation of evoked potentials and EEG phenomena. Physiol. Rev. 65, 37–100 10.1007/s12021-011-9111-4 3880898

[B54] Moore B. C. Glasberg B. R. (1983). Masking patterns for synthetic vowels in simultaneous and forward masking. J. Acoust. Soc. Am. 73, 906–917 10.1121/1.2019092 6221041

[B55] Moore B. C. Glasberg B. R. (1987). Formulae describing frequency selectivity as a function of frequency and level, and their use in calculating excitation patterns. Hear. Res. 28, 209–225 10.1016/0378-5955(87)90050-5 3654390

[B55a] Moore B. C. Gockel H. E. (2011). Resolvability of components in complex tones and implications for theories of pitch perception. Hear. Res. 276, 88–97 10.1016/j.heares.2011.01.003 21236327

[B56] Nondahl D. Shi X. Cruickshanks K. Dalton D. Tweed T. Wiley T. (2009). Notched audiograms and noise exposure history in older adults. Ear Hear 30, 696–703 10.1097/AUD.0b013e3181b1d418 19633561 PMC2811687

[B57] Norena A. J. (2011). An integrative model of tinnitus based on a central gain controlling neural sensitivity. Neurosci. Biobehav. Rev. 35, 1089–1109 10.1016/j.neubiorev.2010.11.003 21094182

[B58] Noreña A. J. Eggermont J. J. (2003). Neural correlates of an auditory afterimage in primary auditory cortex. J. Assoc. Res. Otolaryngol 4, 312–328 10.1007/s10162-002-3039-1 14690050 PMC3202731

[B59] Norena A. J. Eggermont J. J. (2005). Enriched acoustic environment after noise trauma reduces hearing loss and prevents cortical map reorganization. J. Neurosci. 25, 699–705 10.1523/JNEUROSCI.2226-04.2005 15659607 PMC6725313

[B61] Noreña A. J. Farley B. J. (2013). Tinnitus-related neural activity: theories of generation, propagation, and centralization. Hear. Res. 295, 161–171 10.1016/j.heares.2012.09.010 23088832

[B62] Norena A. J. Gourevitch B. Aizawa N. Eggermont J. J. (2006). Spectrally enhanced acoustic environment disrupts frequency representation in cat auditory cortex. Nat. Neurosci. 9, 932–939 10.1038/nn1720 16783369

[B63] Norena A. J. Gourevitch B. Pienkowski M. Shaw G. Eggermont J. J. (2008). Increasing spectrotemporal sound density reveals an octave-based organization in cat primary auditory cortex. J. Neurosci. 28, 8885–8896 10.1523/JNEUROSCI.2693-08.2008 18768682 PMC6670867

[B64] Noreña A. J. Tomita M. Eggermont J. J. (2003). Neural changes in cat auditory cortex after a transient pure-tone trauma. J. Neurophysiol. 90, 2387–2401 10.1152/jn.00139.2003 12773493

[B65] Norena A. Micheyl C. Chery-Croze S. (2000). An auditory negative after-image as a human model of tinnitus. Hear. Res. 149, 24–32 10.1016/S0378-5955(00)00158-1 11033244

[B66] Okamoto H. Kakigi R. Gunji A. Pantev C. (2007). Asymmetric lateral inhibitory neural activity in the auditory system: a magnetoencephalographic study. BMC Neurosci. 8:33 10.1186/1471-2202-8-33 17509141 PMC1884167

[B67] Oxenham A. J. Simonson A. M. (2006). Level dependence of auditory filters in nonsimultaneous masking as a function of frequency. J. Acoust. Soc. Am. 119, 444–453 10.1121/1.2141359 16454299 PMC1752201

[B68] Palmer A. Shamma S. (2004). Physiological representations of speech, in Speech Processing in the Auditory System Springer Handbook of Auditory Research, eds Greenberg S. Ainsworth W. A. Popper A. N. Fay R. R. (New York, NY: Springer), 163–230 Available online at: http://www.springerlink.com/content/g764412j03104148/abstract/ [Accessed July 27, 2012]. 10.1007/0-387-21575-1_4

[B69] Pantev C. Wollbrink A. Roberts L. E. Engelien A. Lütkenhöner B. (1999). Short-term plasticity of the human auditory cortex. Brain Res. 842, 192–199 10.1016/S0006-8993(99)01835-1 10526109

[B70] Parra L. C. Pearlmutter B. A. (2007). Illusory percepts from auditory adaptation. J. Acoust. Soc. Am. 121, 1632–1641 10.1121/1.2431346 17407900

[B71] Pettet M. W. Gilbert C. D. (1992). Dynamic changes in receptive-field size in cat primary visual cortex. Proc. Natl. Acad. Sci. U.S.A. 89, 8366–8370 1518870 10.1073/pnas.89.17.8366 PMC49919

[B72] Pienkowski M. Munguia R. Eggermont J. J. (2013). Effects of passive, moderate-level sound exposure on the mature auditory cortex: spectral edges, spectrotemporal density, and real-world noise. Hear. Res. 296, 121–130 10.1016/j.heares.2012.11.006 23154196

[B73] Poon P. W. Brugge J. F. (1993). Sensitivity of auditory nerve fibers to spectral notches. J. Neurophysiol. 70, 655–666 8410165 10.1152/jn.1993.70.2.655

[B74] Quiroga R. Q. Nadasdy Z. Ben Shaul Y. (2004). Unsupervised spike detection and sorting with wavelets and superparamagnetic clustering. Neural Comput. 16, 1661–1687 10.1162/089976604774201631 15228749

[B75] Rabinowitz N. C. Willmore B. D. B. Schnupp J. W. H. King A. J. (2011). Contrast gain control in auditory cortex. Neuron 70, 1178–1191 10.1016/j.neuron.2011.04.030 21689603 PMC3133688

[B76] Rabinowitz N. C. Willmore B. D. B. Schnupp J. W. H. King A. J. (2012). Spectrotemporal contrast kernels for neurons in primary auditory cortex. J. Neurosci. 32, 11271–11284 10.1523/JNEUROSCI.1715-12.2012 22895711 PMC3542625

[B77] Rabinowitz P. M. Galusha D. Slade M. D. Dixon-Ernst C. Sircar K. D. Dobie R. A. (2006). Audiogram notches in noise-exposed workers. Ear Hear 27, 742–750 10.1097/01.aud.0000240544.79254.bc 17086083

[B77a] Rajan R. (2001). Plasticity of excitation and inhibition in the receptive field of primary auditory cortical neurons after limited receptor organ damage. Cereb. Cortex 11, 171–182 10.1121/1.381276 11208672

[B78] Rajan R. (1998). Receptor organ damage causes loss of cortical surround inhibition without topographic map plasticity. Nat. Neurosci. 1, 138–143 10.1038/388 10195129

[B79] Rajan R. Irvine D. R. Wise L. Z. Heil P. (1993). Effect of unilateral partial cochlear lesions in adult cats on the representation of lesioned and unlesioned cochleas in primary auditory cortex. J. Comp. Neurol 338, 17–49 10.1002/cne.903380104 8300898

[B80] Ramachandran V. S. Gregory R. L. (1991). Perceptual filling in of artificially induced scotomas in human vision. Nature 350, 699–702 10.1038/350699a0 2023631

[B81] Ratliff F. Hartline H. K. (1959). The responses of Limulus optic nerve fibers to patterns of illumination on the receptor mosaic. J. Gen. Physiol. 42, 1241–1255 13664924 10.1085/jgp.42.6.1241 PMC2194959

[B82] Recio A. Rhode W. S. (2000). Representation of vowel stimuli in the ventral cochlear nucleus of the chinchilla. Hear. Res. 146, 167–184 10.1016/S0378-5955(00)00111-8 10913893

[B83] Rhode W. S. Greenberg S. (1994). Lateral suppression and inhibition in the cochlear nucleus of the cat. J. Neurophysiol. 71, 493–514 8176421 10.1152/jn.1994.71.2.493

[B84] Robertson D. Irvine D. R. (1989). Plasticity of frequency organization in auditory cortex of guinea pigs with partial unilateral deafness. J. Comp. Neurol. 282, 456–471 10.1002/cne.902820311 2715393

[B85] Rutkowski R. G. Weinberger N. M. (2005). Encoding of learned importance of sound by magnitude of representational area in primary auditory cortex. Proc. Natl. Acad. Sci. U.S.A. 102, 13664–13669 10.1073/pnas.0506838102 16174754 PMC1200094

[B86] Sachs M. B. Young E. D. (1979). Encoding of steady-state vowels in the auditory nerve: representation in terms of discharge rate. J. Acoust. Soc. Am. 66, 470–479 512208 10.1121/1.383098

[B87] Sadagopan S. Wang X. (2010). Contribution of inhibition to stimulus selectivity in primary auditory cortex of awake primates. J. Neurosci. 30, 7314–7325 10.1523/JNEUROSCI.5072-09.2010 20505098 PMC3842484

[B88] Shamma S. A. (1985). Speech processing in the auditory system. II: lateral inhibition and the central processing of speech evoked activity in the auditory nerve. J. Acoust. Soc. Am. 78, 1622–1632 3840813 10.1121/1.392800

[B89] Shamma S. A. Micheyl C. (2010). Behind the scenes of auditory perception. Curr. Opin. Neurobiol. 20, 361–366 10.1016/j.conb.2010.03.009 20456940 PMC2901988

[B90] Shamma S. A. Symmes D. (1985). Patterns of inhibition in auditory cortical cells in awake squirrel monkeys. Hear. Res. 19, 1–13 10.1016/0378-5955(85)90094-2 4066511

[B91] Silkes S. M. Geisler C. D. (1991). Responses of “lower-spontaneous-rate” auditory-nerve fibers to speech syllables presented in noise. I: General characteristics. J. Acoust. Soc. Am. 90, 3122–3139 10.1121/1.401421 1787250

[B91a] Small A. M. Daniloff R. G. (1967). Pitch of noise bands. J. Acoust. Soc. Am. 41, 506–512 10.1121/1.1910361 6040810

[B92] Steinschneider M. Tenke C. E. Schroeder C. E. Javitt D. C. Simpson G. V. Arezzo J. C. (1992). Cellular generators of the cortical auditory evoked potential initial component. Electroencephalogr. Clin. Neurophysiol. 84, 196–200 10.1016/0168-5597(92)90026-8 1372236

[B93] Suga N. (1995). Sharpening of frequency tuning by inhibition in the central auditory system: tribute to Yasuji Katsuki. Neurosci. Res. 21, 287–299 10.1016/0168-0102(94)00868-G 7777219

[B94] Suga N. Zhang Y. Yan J. (1997). Sharpening of frequency tuning by inhibition in the thalamic auditory nucleus of the mustached bat. J. Neurophysiol. 77, 2098–2114 10.1016/0168-0102(94)00868-G 9114258

[B95] Szymanski F. D. Rabinowitz N. C. Magri C. Panzeri S. Schnupp J. W. H. (2011). The laminar and temporal structure of stimulus information in the phase of field potentials of auditory cortex. J. Neurosci. 31, 15787–15801 10.1523/JNEUROSCI.1416-11.2011 22049422 PMC6623019

[B98] Ulanovsky N. Las L. Farkas D. Nelken I. (2004). Multiple time scales of adaptation in auditory cortex neurons. J. Neurosci. 24, 10440–10453 10.1523/JNEUROSCI.1905-04.2004 15548659 PMC6730303

[B99] Valentine P. A. Eggermont J. J. (2004). Stimulus dependence of spectro-temporal receptive fields in cat primary auditory cortex. Hear. Res. 196, 119–133 10.1016/j.heares.2004.05.011 15464309

[B100] Viemeister N. F. (1988). Intensity coding and the dynamic range problem. Hear. Res. 34, 267–274 10.1016/0378-5955(88)90007-X 3170367

[B101] Von Bekesy G. (1967). Mach band type lateral inhibition in different sense organs. J. Gen. Physiol. 50, 519–532 11526844 10.1085/jgp.50.3.519 PMC2225686

[B102] Von Bekesy G. (1969a). Inhibition as an important part of sensory perception. Laryngoscope 79, 1366–1386 10.1288/00005537-196908000-00002 5806985

[B103] Von Bekesy G. (1969b). Similarities of inhibition in the different sense organs. Am. Psychol. 24, 707–719 5810471 10.1037/h0027934

[B104] Wallace M. N. Rutkowski R. G. Palmer A. R. (2000). Identification and localisation of auditory areas in guinea pig cortex. Exp. Brain Res. 132, 445–456 10.1007/s002210000362 10912825

[B105] Wang J. McFadden S. L. Caspary D. Salvi R. (2002). Gamma-aminobutyric acid circuits shape response properties of auditory cortex neurons. Brain Res. 944, 219–231 10.1016/S0006-8993(02)02926-8 12106684

[B106] Watkins P. V. Barbour D. L. (2008). Specialized neuronal adaptation for preserving input sensitivity. Nat. Neurosci. 11, 1259–1261 10.1038/nn.2201 18820690

[B107] Watkins P. V. Barbour D. L. (2011). Level-tuned neurons in primary auditory cortex adapt differently to loud versus soft sounds. Cereb. Cortex 21, 178–190 10.1093/cercor/bhq079 20457692 PMC3000570

[B108] Watt A. J. Desai N. S. (2010). Homeostatic Plasticity and STDP: keeping a neuron's cool in a fluctuating world. Front. Synaptic. Neurosci. 2:5 10.3389/fnsyn.2010.00005 21423491 PMC3059670

[B109] Wehr M. Zador A. M. (2005). Synaptic mechanisms of forward suppression in rat auditory cortex. Neuron 47, 437–445 10.1016/j.neuron.2005.06.009 16055066

[B110] Wiegrebe L. Kössl M. Schmidt S. (1996). Auditory enhancement at the absolute threshold of hearing and its relationship to the Zwicker tone. Hear. Res. 100, 171–180 10.1016/0378-5955(96)00111-6 8922992

[B111] Wu G. K. Arbuckle R. Liu B.-H. Tao H. W. Zhang L. I. (2008). Lateral sharpening of cortical frequency tuning by approximately balanced inhibition. Neuron 58, 132–143 10.1016/j.neuron.2008.01.035 18400169 PMC2447869

[B112] Yang L. Pollak G. D. Resler C. (1992). GABAergic circuits sharpen tuning curves and modify response properties in the mustache bat inferior colliculus. J. Neurophysiol. 68, 1760–1774 1479443 10.1152/jn.1992.68.5.1760

[B113] Yost W. (1986). Processing of complex signals and the role of inhibition, in Auditory Frequency Selectivity (New York, NY: Plenum Press), 361–370 10.1007/978-1-4613-2247-4_39

[B114] Zhang L. I. Tan A. Y. Y. Schreiner C. E. Merzenich M. M. (2003). Topography and synaptic shaping of direction selectivity in primary auditory cortex. Nature 424, 201–205 10.1038/nature01796 12853959

[B115] Zwicker E. (1964). “Negative Afterimage” in hearing. J. Acoust. Soc. Am. 36, 2413–2415 10.1121/1.1919373 5045252

